# 4-[(Dieth­oxy­phosphino­yl)meth­yl]benzoic acid

**DOI:** 10.1107/S1600536811008282

**Published:** 2011-03-09

**Authors:** S. Karthikeyan, K. Sethusankar, Ganesan Gobi Rajeshwaran, Arasambattu K. Mohanakrishnan

**Affiliations:** a Department of Physics, RKM Vivekananda College (Autonomous), Chennai 600 004, India; b Department of Organic Chemistry, University of Madras, Maraimalai Campus, Chennai 600 025, India

## Abstract

In the title compound, C_12_H_17_N_2_O_5_P, the phospho­nate group is almost orthogonal to both the ethyl groups, with a dihedral angle of 83.75 (11)°. In the crystal, mol­ecules are linked into centrosymmetric dimers *via* pairs of O—H⋯O hydrogen bonds with an *R*_2_^2^(20) graph-set motif. The crystal structure is further consolidated by weak C—H⋯π inter­actions.

## Related literature

For applications of phospho­nate derivatives, see: Hirschmann *et al.* (1994 ▶). For related structures, see: An *et al.* (2008 ▶); Chen *et al.* (2008 ▶). For graph-set motifs, see: Bernstein *et al.* (1995 ▶).
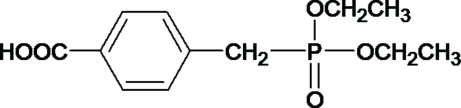


## Experimental

### 

#### Crystal data

C_12_H_17_O_5_P
*M* *_r_* = 272.23
Monoclinic, 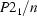

*a* = 9.6505 (5) Å
*b* = 12.1706 (6) Å
*c* = 11.8156 (6) Å
β = 108.926 (2)°
*V* = 1312.74 (12) Å^3^
*Z* = 4
Mo *K*α radiation
μ = 0.22 mm^−1^
*T* = 293 K
0.23 × 0.20 × 0.20 mm

#### Data collection

Bruker SMART APEXII area-detector diffractometer
13181 measured reflections
2960 independent reflections
2441 reflections with *I* > 2σ(*I*)
*R*_int_ = 0.025

#### Refinement

*R*[*F* ^2^ > 2σ(*F* ^2^)] = 0.035
*wR*(*F* ^2^) = 0.106
*S* = 1.04
2960 reflections
165 parameters
H-atom parameters constrained
Δρ_max_ = 0.31 e Å^−3^
Δρ_min_ = −0.22 e Å^−3^

### 

Data collection: *APEX2* (Bruker, 2008) ▶; cell refinement: *SAINT* (Bruker, 2008) ▶; data reduction: *SAINT* ▶; program(s) used to solve structure: *SHELXS97* (Sheldrick, 2008 ▶); program(s) used to refine structure: *SHELXL97* (Sheldrick, 2008 ▶); molecular graphics: *ORTEP-3* (Farrugia, 1997 ▶); software used to prepare material for publication: *SHELXL97*, *PARST* (Nardelli, 1983 ▶) and *PLATON* (Spek, 2009 ▶).

## Supplementary Material

Crystal structure: contains datablocks global, I. DOI: 10.1107/S1600536811008282/pv2396sup1.cif

Structure factors: contains datablocks I. DOI: 10.1107/S1600536811008282/pv2396Isup2.hkl

Additional supplementary materials:  crystallographic information; 3D view; checkCIF report

## Figures and Tables

**Table 1:** Hydrogen-bond geometry (Å, °) *Cg*1 is the centroid of the C2–C7 benzene ring.

*D*—H⋯*A*	*D*—H	H⋯*A*	*D*⋯*A*	*D*—H⋯*A*
O4—H4*A*⋯O1^i^	0.82	1.87	2.644 (2)	158
C12—H12*C*⋯*Cg*1^ii^	0.96	2.97	3.853 (2)	153

Symmetry codes: (i) 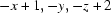; (ii) 
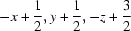
.

**Fig. 1.**
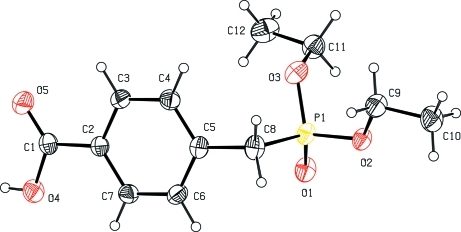
The molecular structure of the title compound with the atom numbering scheme, displacement ellipsoids are drawn at 30% probability level. H atoms are represented by small spheres of arbitary radius.

**Fig. 2.**
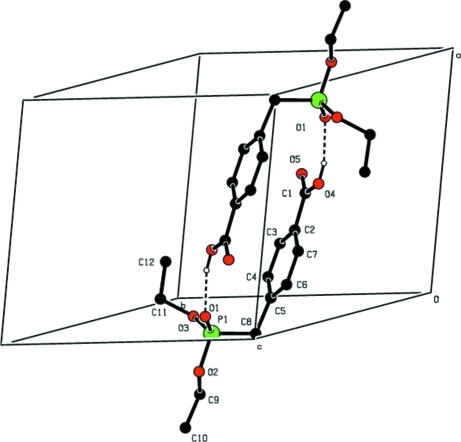
A stereo view of the unit cell of the title compound, showing the dimer formed by H-bonding in R22(20) graph set motif along a axis.

## supplementary crystallographic information

### Comment

Phosphonates have also been used as enzyme inhibitors, anti-HIV agents and haptens for catalytic antibodies (Hirschmann *et al.*, 1994). In this regard, the preparation of various phosphonates with a diversity of structures is highly desirable for drug discovery and medicinal chemistry.

In the title compound (Fig. 1), the phosphonate group is almost orthogonal to the diethyl group with a dihedral angle of 83.75 (11)°. The carboxybenzene ring is essentially planar with the maximum deviation of atom C5 being 0.047 (1) Å. The atom C8 is significantly out of the plane formed by the benzene ring atoms C2/C3/C4/C5/C6/C7; the deviation being 0.108 (2) Å. In addition, the atom C1 of the carboxyl group is 0.095 (2) Å out of the plane of the same benzene ring. The dihedral angle between the benzene ring and the carboxyl group is 3.83 (16)°.

In the crystal, molecules are linked into centrosymmetric dimers *via* pairs of O–H···O hydrogen bonds with a *R*^2^_2_(20) graph set motif (Bernstein, *et al.*, 1995). The crystal structure is further stabilized by C–H···π interaction, where *Cg*1 is the centroid of the benzene ring (C2/C3/C4/C5/C6/C7).

### Experimental

To a solution of 4-(bromomethyl)benzoic acid (1 mmol) and triethylphosphite (1.1 mmol) in dry dichloromethane (10 ml) at room temperature, ZnBr_2_ (0.2 mmol) was added and allowed to stir for 2 h under N_2_. After consumption of 4-(bromomethyl)benzoic acid (monitored by TLC) volatile components were removed under vacuo. The residual mass was poured over crushed ice (200 g) containing conc. HCl (5 ml). The precipitated solid was filtered, washed with water and dried to give crude phosphonate ester. The crude product was purified by flash column chromatography to provide the title compound which was recrystalized from 20% ethylacetate in pure hexane.

### Refinement

Hydrogen atoms were placed in calculated positions with C—H = 0.93 - 0.97Å and O—H = 0.82 Å; refined in the riding model with fixed isotropic displacement parameters: *U*_iso_(H) = 1.5 *U*_eq_(methyl C and O) and *U*_iso_(H) = 1.2 *U*_eq_(C) for other groups.

### Crystal data

**Table:**

C_12_H_17_O_5_P	*F*(000) = 576
*M**_r_* = 272.23	*D*_x_ = 1.377 Mg m^−^^3^
Monoclinic, *P*2_1_/*n*	Mo *K*α radiation, λ = 0.71073 Å
Hall symbol: -P 2yn	Cell parameters from 2960 reflections
*a* = 9.6505 (5) Å	θ = 1.0–25.0°
*b* = 12.1706 (6) Å	µ = 0.22 mm^−^^1^
*c* = 11.8156 (6) Å	*T* = 293 K
β = 108.926 (2)°	Block, colourless
*V* = 1312.74 (12) Å^3^	0.23 × 0.20 × 0.20 mm
*Z* = 4	

### Data collection

**Table:**

Bruker SMART APEXII area-detector diffractometer	2441 reflections with *I* > 2σ(*I*)
Radiation source: fine-focus sealed tube	*R*_int_ = 0.025
graphite	θ_max_ = 27.3°, θ_min_ = 2.4°
ω scans	*h* = −12→12
13181 measured reflections	*k* = −15→15
2960 independent reflections	*l* = −15→15

### Refinement

**Table:**

Refinement on *F*^2^	Primary atom site location: structure-invariant direct methods
Least-squares matrix: full	Secondary atom site location: difference Fourier map
*R*[*F*^2^ > 2σ(*F*^2^)] = 0.035	Hydrogen site location: inferred from neighbouring sites
*wR*(*F*^2^) = 0.106	H-atom parameters constrained
*S* = 1.04	*w* = 1/[σ^2^(*F*_o_^2^) + (0.0578*P*)^2^ + 0.272*P*] where *P* = (*F*_o_^2^ + 2*F*_c_^2^)/3
2960 reflections	(Δ/σ)_max_ = 0.001
165 parameters	Δρ_max_ = 0.31 e Å^−^^3^
0 restraints	Δρ_min_ = −0.22 e Å^−^^3^

### Special details

**Table:**

Geometry. All e.s.d.'s (except the e.s.d. in the dihedral angle between two l.s. planes) are estimated using the full covariance matrix. The cell e.s.d.'s are taken into account individually in the estimation of e.s.d.'s in distances, angles and torsion angles; correlations between e.s.d.'s in cell parameters are only used when they are defined by crystal symmetry. An approximate (isotropic) treatment of cell e.s.d.'s is used for estimating e.s.d.'s involving l.s. planes.
Refinement. Refinement of *F*^2^ against ALL reflections. The weighted *R*-factor *wR* and goodness of fit *S* are based on *F*^2^, conventional *R*-factors *R* are based on *F*, with *F* set to zero for negative *F*^2^. The threshold expression of *F*^2^ > σ(*F*^2^) is used only for calculating *R*-factors(gt) *etc*. and is not relevant to the choice of reflections for refinement. *R*-factors based on *F*^2^ are statistically about twice as large as those based on *F*, and *R*- factors based on ALL data will be even larger.

### Fractional atomic coordinates and isotropic or equivalent isotropic displacement parameters (Å^2^)

**Table:**

	*x*	*y*	*z*	*U*_iso_*/*U*_eq_	
C1	0.56048 (16)	−0.02314 (13)	0.81418 (13)	0.0410 (3)	
C2	0.40996 (15)	0.00742 (12)	0.81102 (12)	0.0367 (3)	
C3	0.34611 (17)	0.10233 (13)	0.75114 (14)	0.0430 (3)	
H3	0.3950	0.1433	0.7094	0.052*	
C4	0.21059 (18)	0.13606 (14)	0.75341 (14)	0.0451 (4)	
H4	0.1691	0.2000	0.7135	0.054*	
C5	0.13554 (16)	0.07576 (13)	0.81447 (13)	0.0409 (3)	
C6	0.19754 (18)	−0.02041 (14)	0.87045 (14)	0.0457 (4)	
H6	0.1467	−0.0632	0.9090	0.055*	
C7	0.33374 (18)	−0.05359 (13)	0.86979 (13)	0.0426 (3)	
H7	0.3748	−0.1177	0.9093	0.051*	
C8	−0.00843 (17)	0.11637 (15)	0.82358 (14)	0.0485 (4)	
H8A	−0.0678	0.0540	0.8304	0.058*	
H8B	−0.0611	0.1561	0.7512	0.058*	
C9	−0.24479 (18)	0.29083 (16)	0.86037 (16)	0.0536 (4)	
H9A	−0.2711	0.2456	0.7889	0.064*	
H9B	−0.2059	0.3599	0.8428	0.064*	
C10	−0.3748 (2)	0.31113 (17)	0.89678 (18)	0.0623 (5)	
H10A	−0.4147	0.2423	0.9110	0.094*	
H10B	−0.4470	0.3501	0.8344	0.094*	
H10C	−0.3473	0.3542	0.9687	0.094*	
C11	0.1723 (2)	0.38949 (16)	0.99414 (17)	0.0558 (4)	
H11A	0.1735	0.3721	1.0746	0.067*	
H11B	0.1314	0.4625	0.9740	0.067*	
C12	0.3235 (2)	0.38617 (19)	0.98815 (19)	0.0662 (5)	
H12A	0.3629	0.3135	1.0074	0.099*	
H12B	0.3837	0.4377	1.0443	0.099*	
H12C	0.3217	0.4051	0.9088	0.099*	
O1	0.10809 (12)	0.15721 (11)	1.06524 (9)	0.0519 (3)	
O2	−0.13529 (11)	0.23494 (10)	0.95801 (9)	0.0451 (3)	
O3	0.08356 (12)	0.30987 (10)	0.91015 (10)	0.0502 (3)	
O4	0.60874 (12)	−0.11402 (10)	0.87523 (10)	0.0513 (3)	
H4A	0.6917	−0.1275	0.8744	0.077*	
O5	0.63179 (14)	0.02983 (11)	0.76684 (12)	0.0608 (3)	
P1	0.01894 (4)	0.20435 (4)	0.95001 (3)	0.04038 (14)	

### Atomic displacement parameters (Å^2^)

**Table:**

	*U*^11^	*U*^22^	*U*^33^	*U*^12^	*U*^13^	*U*^23^
C1	0.0400 (8)	0.0442 (8)	0.0372 (7)	0.0009 (6)	0.0103 (6)	−0.0061 (6)
C2	0.0377 (7)	0.0394 (7)	0.0319 (6)	−0.0005 (6)	0.0097 (5)	−0.0043 (6)
C3	0.0455 (8)	0.0437 (9)	0.0428 (8)	0.0007 (7)	0.0183 (6)	0.0053 (6)
C4	0.0467 (8)	0.0460 (9)	0.0420 (8)	0.0080 (7)	0.0134 (7)	0.0067 (6)
C5	0.0366 (7)	0.0496 (9)	0.0349 (7)	−0.0012 (6)	0.0094 (6)	−0.0066 (6)
C6	0.0476 (8)	0.0478 (9)	0.0462 (8)	−0.0064 (7)	0.0215 (7)	0.0007 (7)
C7	0.0486 (8)	0.0392 (8)	0.0404 (8)	0.0031 (6)	0.0149 (6)	0.0035 (6)
C8	0.0349 (8)	0.0628 (11)	0.0454 (8)	−0.0007 (7)	0.0096 (6)	−0.0077 (7)
C9	0.0436 (9)	0.0682 (12)	0.0482 (9)	0.0145 (8)	0.0136 (7)	0.0164 (8)
C10	0.0432 (9)	0.0730 (13)	0.0693 (12)	0.0140 (9)	0.0161 (8)	0.0071 (10)
C11	0.0579 (10)	0.0583 (11)	0.0522 (9)	−0.0065 (8)	0.0193 (8)	−0.0083 (8)
C12	0.0467 (10)	0.0773 (14)	0.0673 (12)	−0.0097 (9)	0.0083 (9)	0.0114 (10)
O1	0.0407 (6)	0.0720 (8)	0.0398 (6)	0.0129 (6)	0.0088 (5)	0.0073 (5)
O2	0.0365 (5)	0.0608 (7)	0.0389 (5)	0.0101 (5)	0.0136 (4)	0.0100 (5)
O3	0.0453 (6)	0.0648 (8)	0.0390 (6)	−0.0090 (5)	0.0115 (5)	0.0004 (5)
O4	0.0428 (6)	0.0574 (7)	0.0518 (6)	0.0115 (5)	0.0128 (5)	0.0075 (5)
O5	0.0500 (7)	0.0632 (8)	0.0783 (9)	0.0047 (6)	0.0333 (6)	0.0109 (7)
P1	0.0314 (2)	0.0556 (3)	0.0334 (2)	0.00373 (16)	0.00944 (15)	0.00211 (16)

### Geometric parameters (Å, °)

**Table:**

C1—O5	1.2049 (19)	C9—C10	1.473 (2)
C1—O4	1.3201 (19)	C9—H9A	0.9700
C1—C2	1.488 (2)	C9—H9B	0.9700
C2—C7	1.381 (2)	C10—H10A	0.9600
C2—C3	1.390 (2)	C10—H10B	0.9600
C3—C4	1.379 (2)	C10—H10C	0.9600
C3—H3	0.9300	C11—O3	1.451 (2)
C4—C5	1.387 (2)	C11—C12	1.484 (3)
C4—H4	0.9300	C11—H11A	0.9700
C5—C6	1.382 (2)	C11—H11B	0.9700
C5—C8	1.511 (2)	C12—H12A	0.9600
C6—C7	1.377 (2)	C12—H12B	0.9600
C6—H6	0.9300	C12—H12C	0.9600
C7—H7	0.9300	O1—P1	1.4708 (11)
C8—P1	1.7867 (16)	O2—P1	1.5666 (11)
C8—H8A	0.9700	O3—P1	1.5654 (12)
C8—H8B	0.9700	O4—H4A	0.8200
C9—O2	1.4558 (18)		
			
O5—C1—O4	123.27 (14)	O2—C9—H9B	110.0
O5—C1—C2	123.67 (15)	C10—C9—H9B	110.0
O4—C1—C2	113.06 (13)	H9A—C9—H9B	108.4
C7—C2—C3	118.85 (14)	C9—C10—H10A	109.5
C7—C2—C1	121.87 (14)	C9—C10—H10B	109.5
C3—C2—C1	119.24 (13)	H10A—C10—H10B	109.5
C4—C3—C2	120.27 (14)	C9—C10—H10C	109.5
C4—C3—H3	119.9	H10A—C10—H10C	109.5
C2—C3—H3	119.9	H10B—C10—H10C	109.5
C3—C4—C5	120.74 (15)	O3—C11—C12	108.69 (15)
C3—C4—H4	119.6	O3—C11—H11A	110.0
C5—C4—H4	119.6	C12—C11—H11A	110.0
C6—C5—C4	118.64 (14)	O3—C11—H11B	110.0
C6—C5—C8	120.56 (15)	C12—C11—H11B	110.0
C4—C5—C8	120.76 (15)	H11A—C11—H11B	108.3
C7—C6—C5	120.77 (14)	C11—C12—H12A	109.5
C7—C6—H6	119.6	C11—C12—H12B	109.5
C5—C6—H6	119.6	H12A—C12—H12B	109.5
C6—C7—C2	120.68 (14)	C11—C12—H12C	109.5
C6—C7—H7	119.7	H12A—C12—H12C	109.5
C2—C7—H7	119.7	H12B—C12—H12C	109.5
C5—C8—P1	111.44 (10)	C9—O2—P1	121.53 (10)
C5—C8—H8A	109.3	C11—O3—P1	123.12 (11)
P1—C8—H8A	109.3	C1—O4—H4A	109.5
C5—C8—H8B	109.3	O1—P1—O3	115.34 (7)
P1—C8—H8B	109.3	O1—P1—O2	108.59 (6)
H8A—C8—H8B	108.0	O3—P1—O2	107.59 (7)
O2—C9—C10	108.36 (14)	O1—P1—C8	115.07 (8)
O2—C9—H9A	110.0	O3—P1—C8	101.85 (8)
C10—C9—H9A	110.0	O2—P1—C8	107.89 (7)
			
O5—C1—C2—C7	−177.82 (15)	C6—C5—C8—P1	89.85 (17)
O4—C1—C2—C7	1.6 (2)	C4—C5—C8—P1	−87.88 (16)
O5—C1—C2—C3	−0.3 (2)	C10—C9—O2—P1	−178.09 (13)
O4—C1—C2—C3	179.10 (13)	C12—C11—O3—P1	113.06 (15)
C7—C2—C3—C4	1.7 (2)	C11—O3—P1—O1	−31.60 (15)
C1—C2—C3—C4	−175.88 (14)	C11—O3—P1—O2	89.74 (13)
C2—C3—C4—C5	−0.4 (2)	C11—O3—P1—C8	−156.94 (13)
C3—C4—C5—C6	−1.7 (2)	C9—O2—P1—O1	175.49 (13)
C3—C4—C5—C8	176.10 (14)	C9—O2—P1—O3	50.02 (14)
C4—C5—C6—C7	2.6 (2)	C9—O2—P1—C8	−59.17 (15)
C8—C5—C6—C7	−175.21 (14)	C5—C8—P1—O1	−54.31 (15)
C5—C6—C7—C2	−1.4 (2)	C5—C8—P1—O3	71.21 (13)
C3—C2—C7—C6	−0.8 (2)	C5—C8—P1—O2	−175.70 (11)
C1—C2—C7—C6	176.70 (13)		

### Hydrogen-bond geometry (Å, °)

**Table:**

*Cg*1 is the centroid of the C2–C7 benzene ring.

**Table:**

*D*—H···*A*	*D*—H	H···*A*	*D*···*A*	*D*—H···*A*
O4—H4A···O1^i^	0.82	1.87	2.644 (2)	158
C12—H12C···Cg1^ii^	0.96	2.97	3.853 (2)	153

Symmetry codes: (i) −*x*+1, −*y*, −*z*+2; (ii) −*x*+1/2, *y*+1/2, −*z*+3/2.
